# The Matron's Department

**Published:** 1905-12-02

**Authors:** 


### THE MATRON'S DEPARTMENT.

### XIV.—SECRETARIAL DUTIES.

Office work is a great strain upon the matron, demanding as it does in the midst of a round of practical duties a period of impossible abstraction. The brain fatigue involved in writing important letters in the midst of constant interruption often produces a painful irritability which settles into a dull sense of being always in arrears, and takes all the zest out of the work. Much can be done by system to ease this strain, but it is idle to pretend that it can be altogether removed.

At least half an hour should be set aside immediately after breakfast for opening letters, however insistent other claims may be. Appointments should be noted in the rough diary kept for that purpose, money enclosures verified and locked away, any urgent letter answered, and the rest set aside to be dealt with later on. The main business of correspondence, entries in books, and accounts, should be reserved for an hour when interruption will be the exception rather than the rule, and this hour is not likely to be found in the morning. It is far better to surrender the mind without reserve during the stress of the day to those who must depend upon the head for directions. In the afternoon the matron cannot avoid being subject to the inroad of visitors, and must be ready to see the visiting surgeons or physicians if they come to the office. But by five or six o'clock outside interruptions have ceased, the great machine within the walls should be working smoothly, and she may hope to secure the undisturbed hour which will make concentration possible. Receipts for money received should be first attended to, and in addition to the amount received the date, name, and address to which the receipt has been sent should be entered on the counterfoil. Applications for forms, inquiries relating to nurses, and requests for appointments should next receive attention, and, if possible, be cleared up each day as they arise. Lastly will come letters demanding consideration. In the event of any letter containing matter which it is thought advisable to bring before the committee, or which involves consultation or inquiry, it is desirable to send a courteous acknowledgment with an intimation that a further reply will follow, for nothing more readily brings discredit upon an institution than undue delay in acknowledging communications received. All important letters should be written in copying ink and an impression taken by means of the copying-press, which should find a place in every office. It is wiser to destroy no letter received on the spur of the moment. Each one should be stamped as attended to with a rubber stamp fitted with movable date. Letters of any importance should find a place in an alphabetically-lettered file kept in a proper case. Re-fills can be obtained at small cost bound in cardboard with straps and labels, and the files should be numbered and stored away when full for reference. It is better to err on the side of keeping too much than keeping too little, and every three or five years the old files can safely be destroyed. But a vast amount of matter relating to daily engagements will pass through the matron's hands which it can serve no good purpose to preserve except for a day or two. It is a good plan to file all such miscellaneous communications together on a spike and destroy what is not wanted once a week, for it is often very puzzling to tell at first sight what will and what will not be wanted again. All receipts should of course be filed away together and even for the smallest payments some receipt should be forthcoming.

It is well not to answer any letter which is rude or worrying on the day when it is received unless it is of an urgent character. Expressions which seem wilfully impertinent on a first reading may appear when read through again to be merely due to ignorance or hurry. In no case should a rude letter affect the character of the reply, unless it be to induce a tone of rather extra courtesy.

We have already recommended that the matron if on her appointment she has no practical knowledge of book-keeping should take a few lessons in the art. It would greatly simplify her difficulties and lend ease and rapidity to the despatch of business. Hardly two hospitals are alike in the character of the book-keeping which falls to the lot of the matron, but initiation into business methods will enable her readily to grasp the system in vogue. In the event of her succeeding to a post where her predecessor has been in the habit of working "by rule of thumb," recourse should be had to the "Uniform System of Accounts,"[1] where specimen pages are given of all necessary books.

It may fall to the lot of the matron to act as secretary to one or other of the committees connected with her work. As secretary it is her duty to send formal notice of the holding of each meeting to every member at least three days before it is held, unless it should happen that the meetings are held on the same day and hour every week The notice should run as follows:—

A meeting of the Committee will be held at on at o'clock, for the purpose of considering •

(Signed) , *Secretary.*

If the meeting is an ordinary one the purpose may be omitted. An agenda paper containing a numbered series of "things to be done" should be laid before the chairman and the members of the committee. The order of business is as follows:—

1. To read and confirm the minutes of previous meeting.
2. To receive financial and other reports (financial reports take precedence).
3. To consider resolutions on business previously arranged.
4. To do any other business.

In the case of an extraordinary meeting the secretary is first called upon to read the notice which convenes it. The secretary is then called upon to read the minutes of the previous meeting, and must take care that these are signed by the chairman after having been adopted. The minutes should contain a concise record of the proceedings. They should begin with stating the date, hour, and place of the meeting, its purpose, the names of members present, and the name of the chairman. They

[1] By Sir Henry Burdett, K.C.B. (Scientific Press).

should contain an exact statement of everything transacted, in the order followed on the occasion, starting with the adoption of the minutes at the previous meeting. They should not contain records of conversation or discussion, although any statement bearing on matters of fact made by the chairman or member may be briefly summarised. After the minutes have been read and signed, the chairman calls upon any person charged with the duty of presenting a report to read it aloud. It must then be seconded, and if no amendment is moved, is put to the meeting and formally adopted. All reports presented must be inserted in full in the minutes, but the secretary need not, unless requested to do so, read them over again at the next meeting when they recur in the minutes. It is usual to pause and inquire of the chairman, "Is it your pleasure that this shall be taken as read?" and, after receiving an affirmative sign, to pass on to the next item. All other business except the reports is brought forward in the first instance by means of a resolution, moved by one member of the committee and seconded by another. A resolution may be tentative, as, "To consider the desirability of ——" or definite, as, "To appoint ——" or "To direct——." The secretary should pass a slip of paper to the mover of the resolution and obtain it in writing, and should adopt the same means of procuring the correct wording of any amendment which may be moved. It is not necessary to record in the minutes any arguments for or against the resolution which may be brought forward. At the conclusion of the discussion it is put to the meeting and either lost, a majority voting against it, or carried. If all vote in favour of it it is carried "unanimously," if carried with none voting against but some abstaining from voting, it is carried "*nemine contradicente*." Where the meeting is divided hands are counted and the numbers should be recorded. It is contrary to etiquette for the secretary to take any part in the discussion, except by express permission of the chairman, should information be required. In case of any obscurity in the notes taken, or any doubtful point occurring, the secretary should forward a rough copy of the minutes to the chairman and obtain his advice before entering them in the minute-book, from which it is very important to exclude any erasures or errors. It may be added that an attendance-book should be provided in the case of regular meetings for the signatures of members attending the committee.

It will more often fall to the lot of the matron to attend the committees of the hospital in her official capacity than to assume the formal duties of secretary. In the event of weekly reports being expected from her she should enter a copy of every report in a book kept for that purpose. She should provide herself for attendance on the board with memoranda containing any figures likely to be required in a concise form and should be always ready to place full information on matters within her department before the governors. If any system of inspection by members of the board is in force the matron may do much to make it a reality by explaining her plans and setting her experience at the service of those whose duty it is to look into the affairs of the institution. She need have no fear of "interference," that bug-bear of the permanent official in presence of the lay governor, if with tact and directness of purpose she studies to give her supervising authority the same means of forming a correct judgment which she herself possesses.

